# Physicochemical and Functional Properties and Storage Stability of Chitosan–Starch Films Containing Micellar Nano/Microstructures with Turmeric and Hibiscus Extracts

**DOI:** 10.3390/ijms241512218

**Published:** 2023-07-30

**Authors:** Liliana Woszczak, Karen Khachatryan, Magdalena Krystyjan, Teresa Witczak, Mariusz Witczak, Dorota Gałkowska, Małgorzata Makarewicz, Gohar Khachatryan

**Affiliations:** 1 Laboratory of Nanomaterials and Nanotechnology, Faculty of Food Technology, University of Agriculture, Balicka Street 122, 30-149 Krakow, Poland; liliana.woszczak@urk.edu.pl (L.W.); karen.khachatryan@urk.edu.pl (K.K.); 2 Department of Carbohydrates Technology and Cereal Processing, Faculty of Food Technology, University of Agriculture, Balicka Street 122, 30-149 Krakow, Poland; magdalena.krystyjan@urk.edu.pl; 3 Department of Engineering and Machinery for Food Industry, University of Agriculture, Balicka Street 122, 30-149 Krakow, Poland; teresa.witczak@urk.edu.pl (T.W.); mariusz.witczak@urk.edu.pl (M.W.); 4 Department of Food Quality Analysis and Assessment, Faculty of Food Technology, University of Agriculture, Balicka Street 122, 30-149 Krakow, Poland; dorota.galkowska@urk.edu.pl; 5 Department of Fermentation Technology and Microbiology, Faculty of Food Technology, University of Agriculture, Balicka Street 122, 30-149 Krakow, Poland; malgorzata.makarewicz@urk.edu.pl

**Keywords:** chitosan, starch, turmeric, hibiscus, biocomposite, micelle

## Abstract

The dynamic development of the food industry and the growing interest of consumers in innovative solutions that increase the comfort and quality of life push the industry towards seeking pioneering solutions in the field of food packaging. Intelligent and active packaging, which affects the quality and durability of food products and allows one to determine their freshness, is still a modern concept. The aim of our study was to obtain two types of films based on chitosan and starch with micellar nanostructures containing extracts from turmeric rhizomes and hibiscus flowers. The presence of spherical nanostructures was confirmed using a scanning electron microscope. The structural and optical properties of the obtained composites were characterised by Fourier-transform infrared (FTIR), UltraViolet-Visible (UV-VIS), and photoluminescence (PL) spectroscopy. Scanning electron microscopy (SEM) analysis confirmed the presence of spherical micellar structures with a size of about 800 nm in the obtained biocomposites. The presence of nano-/microstructures containing extracts affected the mechanical properties of the composites: it weakened the strength of the films and improved their elongation at break (EAB). Films with nano-/microparticles were characterised by a higher water content compared to the control sample and lower solubility, and they showed stronger hydrophilic properties. Preliminary storage tests showed that the obtained biocomposites are sensitive to changes occurring during the storage of products such as cheese or fish. In addition, it was found that the film with the addition of turmeric extract inhibited the growth of microorganisms during storage. The results suggest that the obtained bionanocomposites can be used as active and/or intelligent materials.

## 1. Introduction

Films based on polysaccharides arouse more and more interest both in the world of science and in the food industry [1,2]. Their use can contribute to reducing environmental pollution, limiting the consumption of non-renewable energy sources, or increasing food safety. The use of materials based on polysaccharides brings several benefits resulting from their properties. They are characterised by non-toxicity, biocompatibility, bioadhesiveness, and biodegradability [3,4]. However, despite the many advantages of using polysaccharides alone, there is a growing demand for the development of materials based on natural polymers with functional properties, in particular as carriers of bioactive substances.

Food spoilage is a major public health concern. The study of new technologies to stop or delay food spoilage is a key aspect in nutritional science. Food packaging is designed to protect the contents against various environmental factors, including moisture, light, oxygen, microorganisms, dust, and mechanical stress. Due to growing consumer demand for low-processed food and ready meals, as well as the globalisation of the food industry, there is a need for solutions that ensure the freshness and optimal quality of food over long periods of time, which drives the development of innovative packaging [5]. Increasingly sought-after alternatives are packing materials of natural origin, which could help to reduce the harmful effects on the environment [6]. All these factors are driving the development of innovative packaging to ensure food safety, extended shelf life, and reduced amounts of non-biodegradable waste generated in the process [7,8]. With the expansion of the food packaging market, apart from food quality, increasing attention has been paid to the issue of the environmental impact of the materials used. The use of sustainable solutions for both active ingredients and the packaging matrix, including the use of renewable and biodegradable materials such as biopolymers, has become a priority [9,10]. Packaging has to be active and smart. Polymer nanocomposites have shown great potential in this respect [11,12,13].

Nanotechnology is a field of science that offers practical applications for a variety of sectors, including medicine, biology, pharmacology, agriculture, and food technology [2,14,15]. Nanostructures are designed to encapsulate a wide range of substances to improve the properties and usability of materials while at the same time minimising the negative impact on human health or the environment [16]. Recently, the topic of the application of nanotechnology in medicine and food technology has been generating a fair amount of interest. In-depth research has been conducted to examine the compatibility and ability of nanoparticles to act as controlled and targeted release systems for the delivery of various ingredients. Polymer nanoparticles can modulate the pharmacokinetic properties of active substances due to their subcellular size and affect the compatibility and biodegradability of the polymers used to produce the nanoparticles [17].

Based on their internal structure, polymer nanoparticles can be divided into nanospheres or nanocapsules, which range in size from 10 to 1000 nm. Nanospheres are characterised by a homogeneous, solid matrix structure with one or more target substances uniformly impregnated. Nanocapsules in turn have a core region (either hollow or filled) as well as a shell-like material covering the encapsulated structure [16]. In recent years, due to their core–shell microstructure, polymer nanocapsules have been gaining interest in terms of their potential application for the delivery of bioactive ingredients. The core of the nanocapsule allows the effective increase in the loading capacity of the bioactive ingredient while at the same time reducing the polymer matrix content of the nanoparticles [17]. An advantage offered by the process of encapsulation is the targeted release of bioactive ingredients in food products [18]. Some of the most widely used carriers for encapsulation are liposomes and micelles. The basic constituents of liposomes are lipids and fatty acids, which occur naturally in cell membranes and are biocompatible and biodegradable [19]. Liposomes are colloidal, vesicular structures composed of one or more lipid layers surrounding a varying number of aqueous compartments. The properties of liposomes depend on the composition of lipids, surface charge, and method of preparation. Liposomes can act as carriers for drugs or other macromolecules delivered to the human and animal body, as they simplify the drug delivery process to a specific site. They also form an attractive solution for the food industry as they can be used to encapsulate unstable compounds (e.g., antimicrobials, flavourings, and bioactive substances) to increase their stability [20]. Phospholipid molecules are the main building blocks of liposomes. The most commonly used phospholipids are soy lecithin, egg lecithin, marine lecithin, and milk phospholipids. The main component of phospholipids is glycerol, acting as a backbone for the polar end and fatty acids. Liposome technology is used for a variety of applications, including the production of functional foods, nutraceuticals, cosmetic products, and pharmaceuticals [21]. Micelles, on the other hand, have a unique core–shell structure and can be used for solubilisation, enveloping hydrophobic compounds and improving their bioavailability [22]. The most popular of these are polymeric micelles, which are self-organising systems in amphiphilic polymers. Their structural properties ensure optimal solubilisation of hydrophobic agents encapsulated in a lipophilic core [23].

Curcumin is a natural polyphenolic compound [22] and the most abundant curcuminoid in the turmeric rhizome. It is used as a colouring and flavouring agent in the food industry [24]. Curcumin exhibits antioxidant, anti-inflammatory, anticancer, and antimicrobial properties [22]. It is characterised by low solubility in water, chemical instability, and low bioavailability, and for this reason it needs to be encapsulated in lipids, hydrogels, cyclodextrin compounds, liposomes, or biopolymer particles [25].

Hibiscus is an ornamental flowering shrub, widely cultivated in East Asia. Its flowering period usually lasts five months and its delicate flowers come in a wide range of colours, shapes, and sizes. Hibiscus has high medicinal and industrial value due to its biological properties and nutritional composition [26]. It is a source of such bioactive compounds as polyphenols, carotenoids, ascorbic acid, and tannins, the content of which varies depending on the part of the plant used, as well as climate, plant maturity, and differences in genotypes [27]. It is an industrial plant, cultivated mainly for its antioxidant and antimicrobial properties [28]. Numerous studies have confirmed that it contains potent bioactive compounds with limited stability. For this reason, encapsulation techniques are used in order to increase their stability [29].

The study presented in this paper aimed to obtain chitosan–starch films acting as carriers of micellar nanostructures composed of a core from an ethanol extract of turmeric or hibiscus and an outer lipid layer. The physicochemical and functional properties and storage stability of the films obtained were subsequently examined.

## 2. Results and Discussion

**Figure 1:**
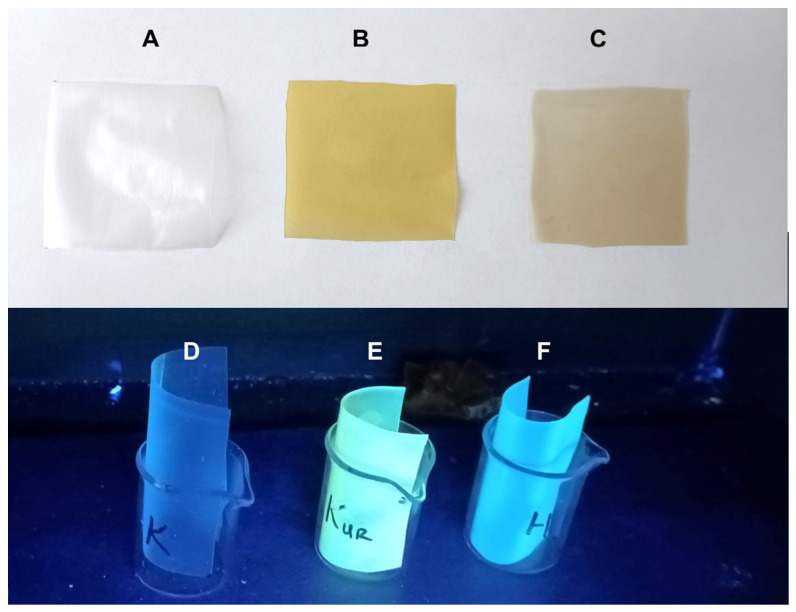
Polysaccharide films in daylight (**A**–**C**) and in UV (365 nm) radiation (**D**–**F**).

Polysaccharide films containing micellar structures with hibiscus and turmeric extracts were successfully obtained by drying the appropriate gels at 25 °C (±2 °C), which is discussed in detail in the methodological part. Figure 1 shows the polysaccharide films in daylight (Figure 1A–C) and in UV (365 nm) radiation (Figure 1D–F). It also shows the film without additives (Figure 1A,D—control) and films containing extracts of hibiscus (Figure 1B,E) and turmeric (Figure 1C,F). 

The morphology, size, and shape of the obtained particles, as well as the structural and optical properties of the obtained composites, were characterised by SEM, FTIR, UV-VIS, and photoluminescence spectroscopy. The functional properties of the obtained films were tested (solubility, degree of swelling, water content, water absorption and water vapour permeability, thickness, transparency, thermal and mechanical properties, contact angle, and colour). Storage tests of the obtained composites were carried out.

### 2.1. Scanning Electron Microscopy (SEM)

**Figure 2:**
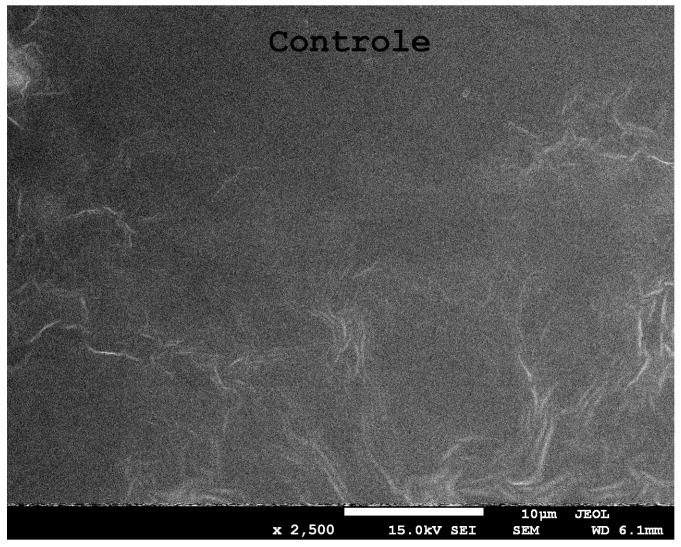
SEM image of the control film at ×2500 magnification.

Scanning electron microscopy (SEM) was used for the visualisation of the surface and morphology of the film, as well as to characterise the resulting micellar nano-/microstructures. Figure 2 shows a microscopic photograph of the control film. The surface of the film is homogeneous, slightly corrugated, with no obvious cracks or pores. This suggests good structural integrity. The plasticiser enhanced the coherence and integrity of the surface structure, resulting in a homogeneous, flexible matrix [30].

**Figure 3:**
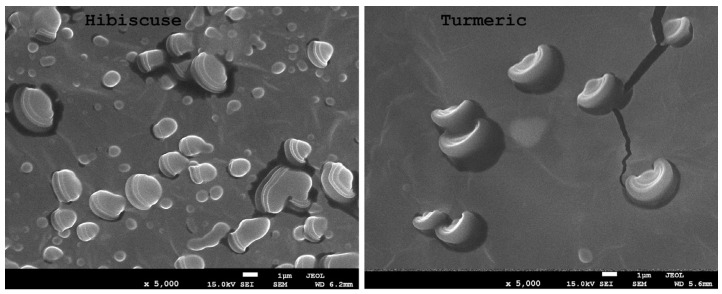
SEM images of the obtained composites (hibiscus and turmeric) ×5000 magnification.

**Figure 4:**
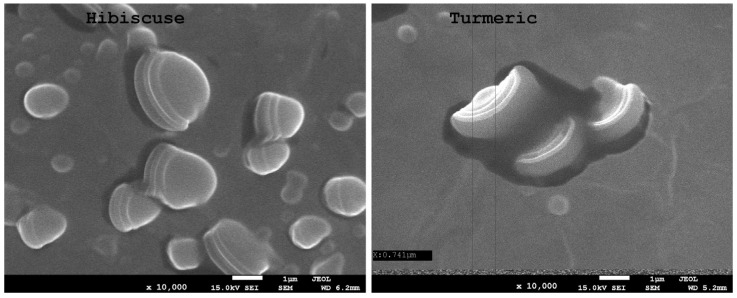
SEM images of the obtained composites (hibiscus and turmeric) ×10,000 magnification.

**Figure 5:**
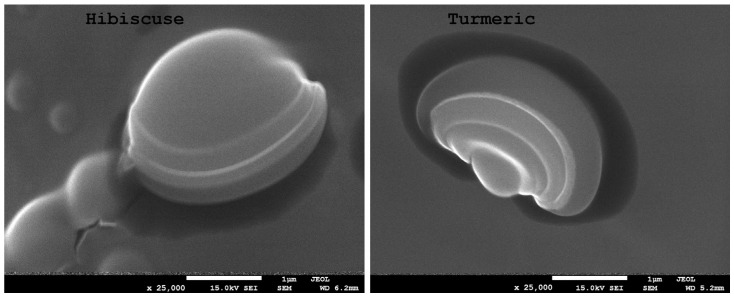
SEM images of the obtained composites (hibiscus and turmeric) ×25,000 magnification.

Figure 3, Figure 4 and Figure 5 show microscopic images of films with nano-/microstructures. In all of the films below, one can observe the presence of spherical nano-/microstructures, distributed in the polysaccharide matrix, ranging in size from 500 to 1500 nm. During electron microscopy imaging, the resulting capsules were bursting in the vacuum bombarded by electrons, which enabled the analysis of their internal structure. The shape of the obtained spherical structures varied slightly depending on the extract used. Figure 5 shows the complex structure of the obtained micelles. In both samples, the capsules consist of a core, a lipid layer (lecithin), and an envelope (polysaccharides). The sizes of the micelle cores with turmeric extracts were significantly smaller than those with hibiscus.

### 2.2. UV-VIS Spectroscopy

**Figure 6:**
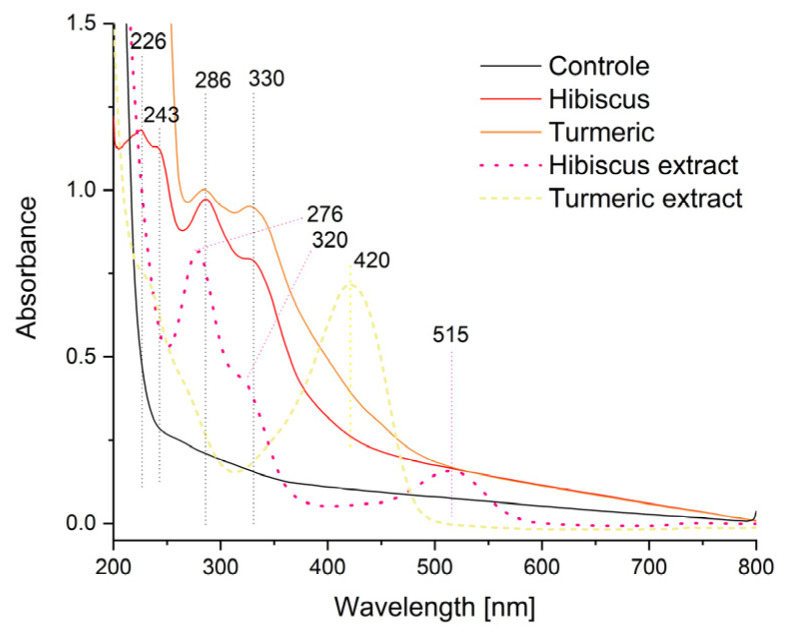
UV-VIS spectra of the control film and the obtained composites.

Figure 6 shows the absorption spectra of the control film and the obtained composites. The control film exhibits no absorbance of radiation in the visible range, while the composites containing nanostructures with extracts show intense absorption bands at wavelengths 330, 286, 243, and 226 nm.

Pure curcumin has characteristic absorption bands at 250 nm and 427 nm, which can be attributed to low-energy π-π* excitation [31]. Hibiscus extracts have two characteristic bands, one of about 328 nm and the other of about 540 nm [32]. The changes in the absorption spectra can be attributed to the functionalisation of the polysaccharide composite with emulsions. Encapsulation causes an increase in absorbance within the 230 to 280 nm range [6,33]. The significant increase in absorbance of the films with micelles indicates that the latter absorb light, which allows for their potential use for the production of the packaging of selected food products in order to protect them from the adverse effects of light radiation.

### 2.3. ATR-FTIR Spectroscopy

**Figure 7:**
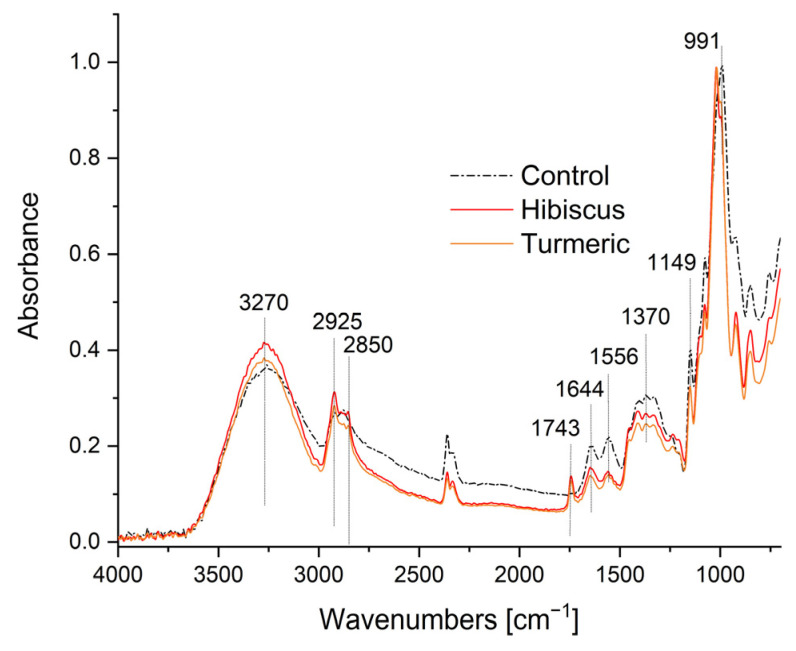
FTIR spectra of the control film and the obtained composites.

Figure 7 shows the FTIR spectra of the control film and the obtained composites. The presence of a broad band at 3270 cm^−1^ in the spectra of the control film and the obtained composites indicates stretching of the OH groups and their overlap with stretching NH groups in the same region. The band at 1556 cm^−1^ indicates the bending of the NH groups (amide II). The band near 1644 cm^−1^ corresponds to carbonyl group stretching vibrations (amide I). The multiple bands in the 1149–950 cm^−1^ range correspond to asymmetric vibrations of C-O-C bridge bonds, asymmetric vibrations of rings, and stretching vibrations of the (C-O) bond. In the spectra of all samples, a band is observed at 2925 and 2850 cm^−1^ from the -CH_2_- and C-H groups in the polysaccharide molecules.

Essentially, the control film and composite spectra are very similar and no significant displacements of bands are observed. Only the differences in the intensity of the absorbance of individual components probably result from intermolecular interactions between the components with different film thicknesses and water content. In the spectra, one can only observe the appearance of a new band at 1743 cm^−1^, corresponding to the carbonyl functional group of triglycerides from the olive oil present in the composites [34]. One can also observe an increase in the intensity of the bands at 2925 cm^−1^ and 2854 cm^−1^ due to the overlapping of symmetric and asymmetric stretching vibration bands of the -CH_2_ and CH_3_ groups from the olive oil [35] with the corresponding bands from the polysaccharides.

### 2.4. Transparency and Colour

Opacity is a property which determines the degree of light impermeability of a material. The higher the value, the lower the transparency of the film and the higher the resistance to UV rays. The nanostructures’ films show lower transparency than the control sample, but as shown by statistical analysis, the difference is insignificant. Light-sensitive foods require packaging with strong UV-blocking properties. However, if the product is to be visible through the packaging, the transparency of the film should be sufficiently high so that the consumer can see the product. The results obtained in this study indicate a high transparency of the film, in comparison with other films described in the literature: based on starch [36], starch and chitosan [37], as well as sodium alginate and chitosan [38].

Table 1 shows the results of the film’s colour measurement. The colour of the material is determined by three parameters. The parameter L* represents lightness (contribution of black or white varying between 0 and 100), where the maximum value indicates the lightest colour and the lowest value is the darkest [6,39]. The parameter L* ranged from 89.49 to 98.72. This means that all films were relatively bright; however, the addition of micellar nanostructures affected the darkening of the surface of the films compared to the control sample. The parameter a* represents the proportion of green or red (negative or positive) in the analysed colour [40,41]. In the control sample and with the addition of curcumin, it indicated the predominance of green shades (the results took a negative value). In contrast, the film with the addition of hibiscus extract showed a predominance of red tones (the value of the parameter a* had a positive value). The parameter b* represents the proportion of blue or yellow (negative or positive) in the analysed colour. As can be seen from the presented data (Table 1), all samples were characterised by the predominance of the yellow shade (the values were positive). The highest values the of b* parameter were characterised by the film with the addition of turmeric extract, because it is a yellow pigment present in the spice turmeric (*Curcuma longa*) [42], often called golden yellow, a naturally occurring polyphenolic compound [43,44]. Our previous studies have confirmed that the yellow pigment of turmeric extract has a decisive effect on the final colour of the composites, regardless of whether the capsules are introduced into a chitosan–alginate matrix [6] or, as in this work, a chitosan–starch matrix. The turmeric extract changes colour under the influence of pH and thus can become an indicator of product freshness [6]. In an acidic environment, films with curcumin show a light-yellow colour, while in an alkaline environment, the colour is intensely red [45,46]. These properties can be effectively used in smart packaging, as curcumin is a good indicator of spoilage in, for example, shrimp, which produce volatile amines during decomposition, causing the pH to change to alkaline and the colour of the film to become red [6,47,48,49]. In an alkaline environment, curcumin’s diketone groups are converted to a keto-enolic form, prompting a spectral shift with a colour transition from yellow to orange/red [45,50]. Thanks to this ability, curcumin can be successfully used as a colourimetric indicator to monitor food spoilage.

The films with the addition of hibiscus extract were characterised by a predominance of red and yellow colour, as it contains a higher quantity of pigments, mainly anthocyanins, which are a natural pigment soluble in water. This pigment changes colour when exposed to different values of pH [51,52]. For example, chitosan/PVA composites containing anthocyanin extracted from purple cabbage were used to indicate the alteration of milk quality from the colour change of the system [53]. In other studies, black carrot [54] and purple sweet potato [55] anthocyanins were applied for monitoring fish freshness.

These observations may therefore suggest that both hibiscus and turmeric extracts have potential uses as natural pH indicators.

### 2.5. Mechanical Properties

The thickness of the obtained films and their mechanical properties are shown in Table 2. Based on the data, it can be seen that the film with hibiscus extract was the thickest (more than twice as thick as the control film). High values of this parameter were also obtained for the film with turmeric extract. Although the same amount of film-forming solution was poured onto the trays, the differences in thickness were statistically significant. This may have been due to the solid content enrichment in the samples [37].

The incorporation of micellar nanostructures into the polysaccharide matrix weakened the strength (TS) of the film, and it also improved its elongation at break (EAB). An increase in the elongation of the chitosan/alginate structure by 99% was observed in the case when turmeric extract was added and by 131% when hibiscus extract was incorporated. The extensibility of films incorporating hibiscus and turmeric extract s were higher than some synthetic materials: polyester (PE), polyvinylidene chloride (PVDC), and low-density polyethylene (LDPE) reported by the Shiku et al. [56].

The strength of the film, on the other hand, decreased 4.9–6.4 times. According to Kumar et al. 2017 [57], this may have been due to the reduction in polymer network continuity and cohesion caused by the introduction of nanoparticles into the matrix.

### 2.6. Solubility and Water Absorption

Table 3 shows the results of the measurements of water content, solubility, and swelling degree of the control film and the films with micellar nanostructures added containing turmeric and hibiscus extracts. The films with nanoparticles added had a higher water content (by approx. 49 and 67%, respectively) compared to the control sample. This indicates that the water encapsulated in the micelles does not evaporate upon drying, confirming the stability of the resulting spherical structures. On the other hand, the solubility of the film with the active substance introduced decreased by 22.6–25.9%, and it was at a similar level of 14.31–14.93% for both samples. It can, therefore, be concluded that the addition of the active substance improved the barrier properties of the film. A low degree of film solubility is a desirable property when the film is to be used for storing food or medicines containing a significant amount of water. Otherwise, it could cause the penetration of film particles into the packaged products. For this reason, the solubility of film components is an important feature that determines its application [37,38]. Statistically significant differences in the degree of swelling between control films and films with nanostructures have also been observed, which may suggest the blocking of certain active groups for water absorption [37].

### 2.7. Water Vapour Barrier Properties

Table 4 shows the water vapour barrier properties of the tested films. Two-factor analysis showed that both RH and film type affected the WVTR and WVP values. In the case of WVTR, the impact of film type was only significantly statistically significant under storage conditions with the lowest relative humidity (RH = 55%), in which case this value was about twice as low as for films with micelles. High relative humidity of the ambient air increased the WVTR significantly relative to RH = 55%. However, it was observed that the value of this parameter did not change as a result of the modification of the film composition.

WVP is a parameter that takes into account the impact of film thickness on water vapour permeability. The addition of micelles to the film increased the WVP value relative to the control sample regardless of storage conditions. The modified films did not differ in terms of WVP value at low RH. In the case of storage under high RH conditions, the film with hibiscus extract exhibited the highest water vapour permeability, and this was correlated with film thickness. It can be observed that the higher RH values did not change the WVP values for individual films, which may suggest that maximum values were reached.

When it comes to water absorption and swelling of the samples, the results obtained can be explained by a change in the water sensitivity of the sample. This was confirmed in works in which, in addition to research related to barrier properties, sorption isotherms were determined [58]. These authors showed that, at low RH values, the process of water vapour sorption through the film played a key role, while values of partial pressure of water vapour above 1300 Pa increased the force driving water vapour through the film barrier. Wiles et al. [59] observed that, at a high partial pressure of water vapour (above 2000 Pa), an increase in moisture sorption in chitosan films can lead to swelling and changes in the structure of the biopolymer. It leads to an increase in the amount of absorbed moisture and loosening of the microstructure of the film. The consequence of this phenomenon is an increase in the stream of water vapour flowing through the matrix as well as a change in their mechanical properties [60,61]. Therefore, when considering the application properties of films based on biopolymers, their water sensitivity should be analysed. The literature data show that chitosan films have lower water vapour permeability in relation to other biopolymer films [62]; therefore, even though the incorporation of micellar nanostructures into the film matrices reduced their barrier properties, they are still an interesting application solution as biodegradable packaging.

### 2.8. Water Contact Angle

The values of the static contact angles of the films against deionised water are listed in Table 5. Due to the fact that the produced films were characterised by different surface structures on both sides, two series of contact angle measurements were carried out, i.e., on the matte surface and on the glossy surface. The values of the contact angles provide information on the wettability of the tested material with a specific liquid. The terms hydrophilicity and hydrophobicity refer to the affinity towards and repellency against water, respectively. In general, they are defined based on the three-phase contact angle. (θ): θ < 90° indicates a hydrophilic surface, and θ > 90° indicated a hydrophobic surface [63]. It should be noted, however, that the contact angle is the combined effect of the affinity of the liquid towards the surface and the physicochemical heterogeneity of the surface [63]. In addition, the contact angle is sensitive to environmental conditions, sample preparation and measurement methods [64]. Thus, it may not be possible to compare the literature data on surface phenomena in the same systems.

The presence of nanoparticles containing hibiscus or turmeric extract in starch–chitosan films resulted in increased hydrophilicity of the films, which was reflected in lower values of the contact angles. This finding is in accordance with the water vapour barrier properties reflected in the WVP values of the films (Table 4). Stanisławska et al. [6] also found that the sodium alginate–chitosan film with curcumin nanocapsules exhibited higher hydrophilicity compared to the control film. In the present study, it was also observed that the matte (slightly rough) surface of the film was slightly less hydrophilic than the glossy (smooth) surface. It can be assumed that the roughness of the matte surface of the film resulted in partial wetting of the surface, with air inclusions between the droplet and solid elements of the film surfaces. Moreover, the different degree of hydrophilicity of both sides of the films could result from the different concentration of nanocapsules in the successive layers of the films, depending on the methods of their production. When the waterdrops deposited on the films were imaged for about 20 s, it was observed that they were absorbed by the films but not expanded on the film surfaces. This proves the strong hydrophilic properties of the films and is in line with the results of measurements of the water vapour barrier properties.

### 2.9. Differential Scanning Calorimetry (DSC)

**Figure 8:**
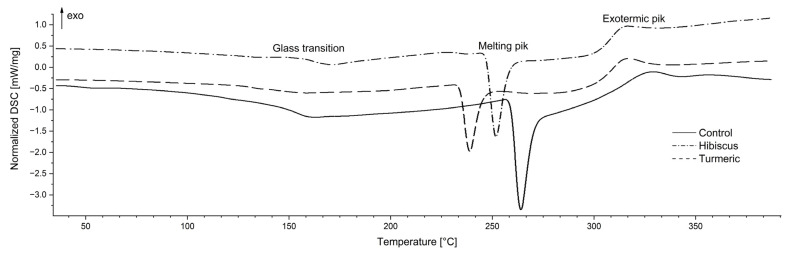
Example thermograms of the examined films.

Table 6 and Table 7 show the results of the DSC analysis of the examined films. Three characteristic phenomena were identified. The first is related to the glass transition phenomenon (Table 7). As can be seen in Figure 8, this transformation is fairly complex. On the one hand, it may be related to the history of the sample and the relaxation phenomenon, and on the other, it may be related to the overlapping of the glass transition phenomenon of the main film components—chitosan and starch. The transition occurs in typical ranges for these substances.

According to Dong et al. [65], the glass transition temperature of chitosan is in the range of 140–150 °C, which is consistent with the values obtained in this study. However, the above-mentioned values are significantly higher than those reported by Jiang et al. [66] obtained for chitosan films, which can in turn be attributed to the presence of starch. As can be seen in Table 6, no significant effect of film modification on the values characterising this phenomenon was found, which also suggests that it is exclusively related to the main components. Another transformation is related to the phenomenon of melting of crystalline structures (Table 6), one of the important characteristics of polysaccharides. Starch consists of two polymers—linear amylose and branched amylopectin. According to Dome et al. [67] the complex of these polysaccharides forms grains/structures that are partially crystalline. The grains contain alternating semi-crystalline and amorphous areas, and the degree of crystallinity depends on the origin and composition of the starch-containing raw material. In turn, in the case of chitin and chitosan, the crystallinity depends on the proportion of different monomers present in chitosan. In this case, the crystallinity is influenced, among others, by the deacetylation process, which may lead to a decrease in chitosan crystallinity, which may be due to the breaking of extensive intermolecular hydrogen bonds present in chitin. In general, chitin has a higher crystallinity than chitosan [68]. The crystallinity of starch–chitosan systems depends on the share of individual polymers. In the study of Lopez et al. [69], the addition of chitosan to corn starch did not have a statistically significant effect on the values of characteristic temperatures, despite a noticeable downward trend. The authors attributed this to the interaction between starch and chitosan molecules, which interrupts the rearrangement of the starch polymer chain. In this work, a statistically significant impact of the modification was found on the values of temperature and enthalpy. The addition of hibiscus had no effect on these parameters and this sample did not differ from the control sample (despite the visible decrease). In turn, the addition of curcumin caused a decrease in the values of all the quantities characterising the melting peak. The decrease in the characteristic temperatures may be caused by the modification of the crystal lattice after the addition of hibiscus/curcumin, which leads to a weakening of the interactions between polysaccharide molecules and, as a result, a reduction in the amount of energy necessary to break them down [69]. In turn, the decrease in enthalpy can be attributed to increased water retention in the system. According to Lopez et al. [69] and Tongdeesoontorn et al. [70], increased interactions between hydrocolloids and starch result in increased water retention and greater water mobility during heating, increased kinetic energy, and decreased enthalpy. This can be confirmed by the increased water content in the tested samples (Table 3). The last peak should be attributed to the phenomenon of polymer breakdown. According to Mathew et al. [71], the exothermic peak starting at 250 °C in chitosan- and starch-based films can be attributed to polymer breakdown, including dehydration of saccharide rings, depolymerisation, and breakdown of acetylated and deacetylated chitosan units. In this case, a change in the peak temperature value following modification was found in both cases analysed, which may suggest compromised thermal stability of the modified polymers.

### 2.10. Microbial Storage Stability

No pathogenic bacteria such as *Salmonella* sp., *Listeria* sp., and *S. aureus* were found in the analysed food products, both fresh and stored.

Based on the results obtained, it can be concluded that the films with hibiscus or curcumin extract had no significant effect on the total bacterial count during storage of cottage cheese (Table 8). Perhaps in this case the concentration of curcumin extract in the tested film was too low to obtain an antibacterial effect. It should be noted that a statistically significant effect was noticed for the foil with curcumin during storage of salmon (Table 8). Admittedly, the total bacterial count increased by one order of magnitude in relation to the number of bacteria determined in the fresh product but was significantly lower when compared to the stored product in the other analysed cases. The total count of *E. coli* bacteria after the storage period was in this case similar to that determined for the fresh product but significantly lower compared to the other variants of the experiment, too (Table 8). The results obtained for the film with curcumin are consistent with our previous studies [6] and with other reports in the literature on the subject [72,73,74,75,76]. Curcumin has been shown to have strong antimicrobial properties against both Gram-positive and Gram-negative bacteria. It has been proven to effectively inhibit biofilm formation by bacteria, including *Escherichia coli*, through interfering with bacterial Quorum Sensing (QS). Curcumin has also been found to exhibit a photodynamic effect by producing cytotoxic reactive oxygen species (ROS), in addition to inhibiting bacterial DNA replication and altering gene expression. It can also damage the bacterial cell membrane, reduce microbial motility, and inhibit cell division and bacterial proliferation [77,78]. It has also been shown that nanoparticles with curcumin exhibit potent antimicrobial properties against S. aureus in periarticular joint infections [79].

### 2.11. Fluorescence Spectroscopy

**Figure 9:**
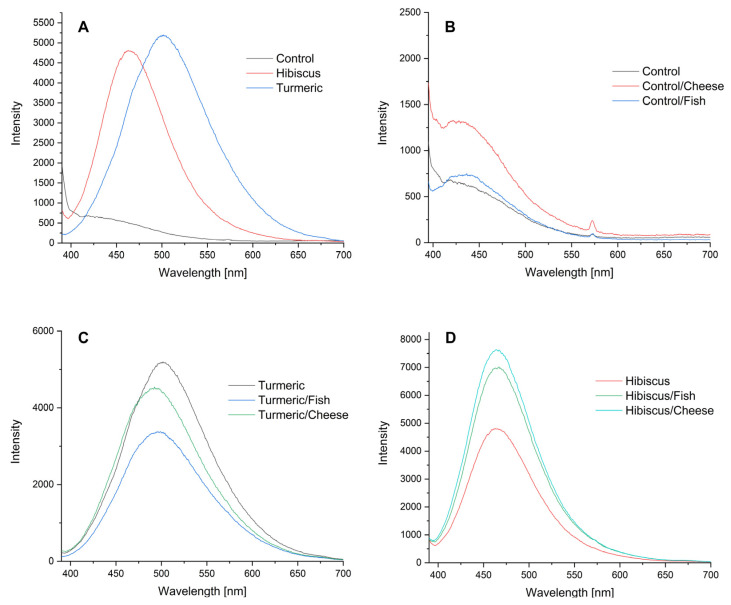
Emission spectra of the films: (**A**)—control film and films containing nanostructures with hibiscus and turmeric extract; (**B**)—control film before and after storage of cheese and fish; (**C**)—film containing nanostructures with turmeric extract before and after storage of cheese and fish; (**D**)—film containing nanostructures with hibiscus extract before and after storage of cheese and fish.

Photoluminescence spectra were measured to verify the optical properties of the obtained films, as well as possible sensitivity to changes during the storage of food (Figure 9).

Figure 9A shows the emission spectra of a starch–chitosan film (control sample) and films containing nanostructures with hibiscus and turmeric extracts. The presence of nanostructures has a significant impact on the emission intensity of the films obtained. An intense band can be observed on the spectra with a maximum at 463 and 501 nm for films with hibiscus and turmeric, respectively. Storage of cheese and fish in control films causes minimal changes in emission properties (Figure 9B). A fairly significant difference is observed in the case of cheese storage, which may be due to the formation of acidic products that cause an increase in chitosan emission intensity.

In the case of films containing extracts, the emission changes depend on the nature of the food product. For composites containing turmeric extracts (Figure 9C), in addition to the change in emission intensity, one can also observe a shift in the emission maximum from 501 nm to 492 nm in the case of cheese storage and 497 nm in the case of fish storage. The changes in intensity may be due to either the presence of acidic or basic compounds formed on the micelle structure or structural changes of the turmeric extract (keto-enol tautomerism).

For composites containing hibiscus extracts (Figure 9D), a significant increase in emission intensity is observed following fish storage (from 4800 to 6950) and cheese storage (up to 7650). Anthocyanins present in the hibiscus extract within the nanostructures are responsible for the emissions. In general, anthocyanins exhibit better solubility in water, so changes in emissions may also result from changes in water content. The intensity of emissions depends on both the concentration and the pH value, as well as the presence of other biological components that can also affect the acid–base balance. The results of the emission tests conducted suggest that the bionanocomposites obtained are sensitive to changes occurring during storage of cheese and fish and could therefore serve as sensors of the freshness of the aforementioned products.

## 3. Materials and Methods

### 3.1. Materials

The following chemical reagents were used to produce the nanocomposites: chitosan (high molecular weight: 310,000–375,000 Da, degree of deacetylation > 75%, from shrimp shells (Sigma-Aldrich, Poznań, Poland)); potato starch, soy lecithin, acetic acid (99%), glycerine (99.5%), and ethanol (96% p.a. grade) were purchased from Pol-Aura (Pol-Aura, Morąg, Poland); extra virgin olive oil, turmeric, and hibiscus were purchased from Agnex (Agnex, Białystok, Poland); deionised water (demineralizer Polwater DL3-150, Labopol-Polwater, Kraków, Poland). None of the chemical reagents had been subjected to prior purification before being used in the experiments.

### 3.2. Methods

To obtain composites containing micellar nanostructures, a starch–chitosan gel was prepared. Then, emulsions containing appropriate extracts, olive oil, and water were prepared. After the emulsion was obtained, lecithin was added to form a micellar structure. The emulsions thus obtained were immediately introduced into the polymer matrix to stabilise the micelles obtained. The resulting gels were dried to obtain the final films. The control film was obtained by drying the gel without the addition of emulsion.

#### 3.2.1. Method of Obtaining Turmeric and Hibiscus Extract

An amount of 150 g of hibiscus and turmeric extracts were prepared. An amount of 30.0 g of ground hibiscus flowers and 30.0 g of ground turmeric rhizome were extracted with ethanol (247.5 mL) using a Soxhlet extractor. The extraction was carried out for 5 h.

#### 3.2.2. Preparation of Turmeric and Hibiscus Nanoemulsions

Two nanoemulsions (10 g each) containing turmeric and hibiscus nanocapsules were prepared by placing 2 mL demineralised water, 5 g turmeric (or hibiscus) extract, and 3 g olive oil in a 25 mL conical flask. The flask was then placed in an ultrasonic bath (2 °C). The mixture was exposed to ultrasound, at 40 kHz, for 15 min. The lecithin suspension (1.25 g in 118.75 mL water) was then added and again exposed to ultrasound for 10 min.

#### 3.2.3. Preparation of Chitosan–Starch Matrix

A suspension of 36 g of potato starch in 1164 mL of water was prepared. The resulting suspension was stirred using a magnetic stirrer at 70 °C, 700 rpm, until a homogeneous 3% potato starch gel was obtained.

Similarly, 450 g of 2% chitosan gel was prepared by weighing out 9 g of chitosan and dissolving it in 441 g of 0.5% acetic acid. The suspension was placed on a magnetic stirrer (700 rpm, temperature: 70 °C) until a homogeneous gel was obtained.

The transparent polysaccharide gels were combined to obtain 1650 g of starch/chitosan gel and homogenised for 10 min using a homogeniser (Polytron PT 2500 E, Kinematica AG, Malters, Switzerland) at 10 °C, 12,000 rpm. The gel obtained was treated as a polymer matrix in the subsequent steps.

#### 3.2.4. Preparation of the Control Film (Control)

A total of 550 g of the previously prepared matrix was weighed out, then 7.5 g of glycerol (used as a plasticiser), 6.2 mL of ethanol, and 125 mL of demineralised water were added to the matrix and mixed using a homogeniser (Polytron PT 2500 E, Kinematica AG, Malters, Switzerland) for 10 min at 10 °C, 12,000 rpm. The clear gel obtained after thoroughly mixing the matrix was poured into four rectangular trays, 150 g each. Once completely dried, a flexible film (control) was obtained.

#### 3.2.5. Preparation of a Film Containing Hibiscus Extract (Hibiscus)

A total of 550 g of the previously prepared matrix was weighed out, then 7.5 g of glycerine (used as plasticiser) and 130 g of hibiscus nanoemulsion (prepared at stage 3.2.2.) were added to the matrix and mixed using a homogeniser (Polytron PT 2500 E, Kinematica AG, Malters, Switzerland) for 10 min at 10 °C, 12,000 rpm. The resulting homogenised mixture was poured into 4 rectangular trays (150 g each). Once completely dried, a flexible film was obtained (labelled Hibiscus).

#### 3.2.6. Preparation of a Film Containing Turmeric Extract (Turmeric)

A total of 550 g of the previously prepared matrix was weighed out, then 7.5 g of glycerine (used as plasticiser) and 130 g of turmeric nanoemulsion (prepared at stage 3.2.2.) were added to the matrix and mixed using a homogeniser (Polytron PT 2500 E, Kinematica AG, Malters, Switzerland) for 10 min at 10 °C, 12,000 rpm. The resulting homogenised mixture was poured into 4 rectangular trays (150 g each). Once completely dried, a flexible film was obtained (labelled Turmeric).

All the films were dried for two days at 25 °C (±2 °C) and approximately 45% humidity. After drying, the films were detached from the trays and placed in tightly sealed string bags until analysis.

#### 3.2.7. SEM Microscopy

The films obtained were analysed in terms of nanoparticle size and morphology using a JEOL 7550 scanning electron microscope (Akishima, Tokyo, Japan). Prior to the measurements, the samples were sprayed (K575X Turbo Sputter Coater, Emitech, Ltd, Kent, UK) with 20 nm Chromium (Cr) to increase the conductivity of the samples.

#### 3.2.8. ATR-FTIR Spectroscopy

The ATR-FTIR spectra of the fabricated composite films were analysed in a wavelength range of 4000–700 cm^−1^ using a MATTSON 3000 FT-IR spectrophotometer (Madison, WI, USA), equipped with a 30SPEC 30 Degree Reflectance accessory (MIRacle ATR, PIKE Technologies Inc, Madison, WI, USA). Measurements were made at a temperature of 25 °C (±2 °C).

#### 3.2.9. UV-VIS Spectroscopy

The UV-VIS absorption spectra of all the prepared films were analysed using a Shimadzu 2101 scanning spectrophotometer (Shimadzu, Kyoto, Japan) in the wavelength range 200–700 nm.

#### 3.2.10. Photoluminescence Spectroscopy

Photoluminescence measurements for the films were carried out at room temperature using a HITACHI F7000 spectrophotometer. The emission spectra of the film and the film coated on food products after a storage test were measured using an excitation wavelength of 380 nm.

#### 3.2.11. Film Opacity

The degree of UV impermeability of the films was measured by exposing the film sample to 600 nm light emitted from a Helios-Gamma 100–240 UV/Vis spectrophotometer [6]. Rectangular film samples were placed directly into the test cell of the spectrophotometer. The empty test cell served as a reference. The opacity (O) of the films was calculated according to the equation:

(1) $$O=A_{600}/x$$

where *A*_600_ is the absorbance at 600 nm and *x* is the film thickness (mm). A higher *O* value indicated a higher degree of opacity/opacification of the sample. The analyses were performed in five replicates.

#### 3.2.12. Surface Colour Analysis

The surface colour was measured with a Konica MINOLTA CM-3500d spectrophotometer (Konica Minolta Inc., Tokyo, Japan) with a ⌀30 mm measuring window, at the following illumination/observation conditions: D65/10°. The results were quantified using the CIE-Lab system. The following parameters were determined: L* (L* = 0 black, L* = 100 white); a*—the proportion of green (a* < 0) or red (a* > 0); and b*—the proportion of blue (b* < 0) or yellow (b* > 0). Measurements were made against a white background of the standard [37]. The experiment was repeated 5 times.

#### 3.2.13. Determination of Water Content, Solubility, and Degree of Swelling

Firstly, 2 × 2 cm squares were cut out of the film samples and weighed using an analytical balance (*m*_1_). The samples were then dried in an oven at 70 °C for 24 h and weighed again (*m*_2_). The squares were placed in beakers containing 30 mL of deionised water, covered and stored for 24 h at room temperature (22 ± 2 °C). The remaining water was removed and the samples were dried on the surface with filter paper and then weighed (*m*_3_). The remaining samples were dried in an oven at 70 °C for 24 h and then weighed (*m*_4_). Three measurements were taken for each sample and the average value of the parameter was determined. These values were then calculated using the following formulae:

(2) $$Water\;content\;\left[\%\right]=\frac{\left(m_{1}-m_{2}\right)}{m_{1}}\text{·}100\%$$

(3) $$Solubility\;\left[\%\right]=\frac{\left(m_{2}-m_{4}\right)}{m_{2}}\text{·}100\%\;$$

(4) $$Degree\;of\;swelling\;\left[\%\right]=\frac{\left(m_{3}-m_{4}\right)}{m_{3}}\text{·}100\%$$

The equation for calculating the degree of swelling was modified according to the method reported by Souza et al. [80], taking into account the dissolved part of the film, so the final dry weight (*m*_4_) was included in the calculations.

#### 3.2.14. Mechanical Tests: Measurement of Film Thickness, Tensile Strength, and Percent Elongation at the Break

Dry films were conditioned in desiccators at 20 °C and 55% relative humidity (RH) by using saturated solutions of magnesium nitrate-6-hydrate for at least 48 h prior to analyses.

##### Thickness Measurement of Composites

The thickness was measured using a screw micrometre (Sylvac SA, Crissier, Switzerland) with an accuracy of 0.001 mm. Each film was measured five times. The result obtained was the arithmetic average of all measurements.

###### Mechanical Properties of Composites

The analysis was performed in accordance with ISO Standards [81]. The films were cut into 35 × 6 mm strips and placed in grips. The initial distance between the grips was 20 mm and the peel rate was 2 mm/min. Tensile strength (TS) was calculated by dividing the maximum force at the break point of the film by its cross-sectional area. The percent elongation at break (EAB) was calculated by dividing the elongation at the break point by the initial measurement length and multiplying the obtained value by 100 [35]. The results reported were the average results of ten repetitions.

#### 3.2.15. Determination of the Wetting Angles

Static water contact angles (WCAs) were measured with a sessile drop technique using a video-based contact angle meter (OCA 25, DataPhysics Instruments GmbH, Filderstadt, Germany) controlled by SCA20 software (Version 4.3, DataPhysics Instruments GmbH, Filderstadt, Germany). The measurements were performed at ambient conditions. A deionised water droplet with a volume of 8.5 µL was deposited onto the film surface using a precision syringe. Both matte and glossy surfaces of the films were evaluated. WCA was calculated by averaging the right and the left contact angles determined at the moment of water droplet deposition. The WCA values were taken from three measurements at different positions of the same sample.

#### 3.2.16. Water Vapour Permeability

The barrier properties for water vapour permeability were determined using a gravimetric method based on ISO 2528:2017 [82]. Glass vessels containing silica gel were covered with the test film, sealed tightly, and placed in desiccators with forced air circulation. Tests were carried out under conditions corresponding to three values of relative humidity determined using saturated salt solutions of MgNO_3_—55% and KCl—84% along with distilled water—100% at 20 °C. In order to determine the increase in weight associated with water vapour transport through the films, the samples were weighed for a specific period of time. The water vapour transmission rate (WVTR) and water vapour permeance (WVP) were determined based on measurements taken in triplicate.

#### 3.2.17. Thermal Properties (DSC)

Approximately 2 mg of the film sample was weighed and sealed into aluminium pans. Subsequently, the samples were heated from 25 °C to 400 °C at a rate of 10 °C/min. The empty pan was used as a reference. The tests were performed with a DSC 204F1 Phoenix differential scanning calorimeter (Netzsch, Germany). The characteristic transition temperatures T_ong_ (onset of glass transition), T_midg_ (midpoint of glass transition), T_infg_ (inflexion point of glass transition), Tendg (endpoint of glass transition), T_onm_ (onset of melting), T_pm_ (peak of melting), T_endm_ (endpoint of melting), T_pe_ (peak of exothermic transition), the change in heat capacity ΔC_pg_ (glass transition), and melting heat ΔH_m_ (melting) were determined using Proteus Analysis software (NETZSCH DSC 204 F1, Selb, Germany). The analyses were performed finally in five replicates.

#### 3.2.18. Microbial Storage Stability

The potential antimicrobial properties of the films tested were verified during cold storage of cottage cheese and Atlantic salmon. Three randomly selected unit packs of each of the above-mentioned products were purchased at a retail outlet. Samples for the test were prepared as follows. A 20 mm × 20 mm hole was cut in the cap of a sterile polystyrene container with a scalpel, and 10 g of the cheese or salmon (collected from the central part of the product) was placed in the container. The test or control film was then placed on top of the open container and the whole setup was sealed with the cap using the pre-cut hole. The reference sample was the container without the hole in the cap. All samples were prepared in triplicate. The samples were refrigerated for 5 days. Microbiological analysis was carried out before placing the food in the containers and after the storage period. Homogenates and decimal dilutions were prepared from fresh or stored products, and then the total bacterial count (BD Plate Count Agar, Argenta), the *Escherichia coli* count (Selective *E. coli*/Coliform Agar, Argenta), and cultures were performed for selected pathogenic microorganisms, including *Salmonella* (*Brilliance Salmonella*, Argenta and XLD Biomaxima), *Listeria* (Chromogenic *Listeria* Agar ISO, Argenta), and *Staphylococcus aureus* (Brilliance Staph24, Argenta). After the storage period, the total bacterial count and the *E. coli* count were determined in the products (using the media indicated above) in a similar manner. The results were given in cfu/g of the product. All analyses were performed in three replicates. The results obtained were subjected to one-way analysis of variance (ANOVA).

#### 3.2.19. Statistical Analysis

A one- and two-way analysis of variance was performed with the Statistica 9.0 software (Statsoft, Tulsa, OK, USA).

## 4. Conclusions

For the purpose of the presented study, starch–chitosan biocomposites containing multilayer micellar nano-/microstructures with hibiscus and turmeric extracts were obtained. Electron microscopy analysis confirmed the presence in the obtained biocomposites of spherical micellar structures ranging between 500 and 1500 nm in size. The presence of nano-/microstructures containing hibiscus or turmeric extracts affected the parameters of the obtained composites: it weakened the strength of the films but at the same time improved their elongation at break (EAB). The films with nano-/microparticles exhibited higher water content compared to the control sample, while the solubility of the films was found to be lower as a result of the introduction of the active substance. The films exhibited stronger hydrophilic properties as measured by water contact angle. What was also observed was an increase in the light absorbance of the films with the added micelles, which makes them potentially suitable for application in the packaging of selected food products in order to protect them from the adverse effects of light. FTIR spectroscopy demonstrated that functionalisation does not lead to chemical interactions with polymers and therefore does not disrupt their structure. Preliminary storage tests showed that the obtained biocomposites are sensitive to changes occurring during the storage of such products as cheese and fish. In addition, the film with added turmeric extract was found to inhibit the proliferation of microorganisms during storage. The results suggest that the bionanocomposites obtained can be used as active and/or smart materials. These can potentially find use as freshness sensors for food products, but further research is needed to test their effectiveness on other food products.

## Figures and Tables

**Table 1:** Opacity and colour parameters of the film surface.

Sample	O (−)	L*	a*	b*
Control	4.0 ± 0.5 ^a^	98.72 ± 0.06 ^a^	−0.08 ± 0.01 ^b^	2.98 ± 0.04 ^c^
Hibiscus	4.4 ± 0.1 ^a^	89.49 ± 0.16 ^c^	0.52 ± 0.04 ^a^	20.58 ± 0.26 ^b^
Turmeric	4.6 ± 0.2 ^a^	92.38 ± 0.49 ^b^	−2.69 ± 0.13 ^c^	31.29 ± 2.62 ^a^

Differences between the values marked with the same letters in individual columns are non-significant at a 0.05 level of confidence.

**Table 2:** Thickness and mechanical parameters of the films.

Sample	Thickness (mm)	TS (MPa)	EAB (%)
Control	0.094 ± 0.014 ^c^	14.68 ± 2.48 ^a^	47.05 ± 7.63 ^b^
Hibiscus	0.198 ± 0.010 ^a^	2.34 ± 0.26 ^b^	108.77 ± 7.79 ^a^
Turmeric	0.155 ± 0.010 ^b^	3.00 ± 0.54 ^b^	93.74 ± 8.84 ^a^

TS—tensile strength, EAB—elongation at break. Differences between the values marked with the same letters in individual columns are non-significant at a 0.05 level of confidence.

**Table 3:** Solubility and water absorption of the films.

Sample	Water Content (%)	Solubility (%)	Swelling Degree (%)
Control	16.57 ± 0.64 ^b^	19.30 ± 1.88 ^a^	215 ± 7.41 ^a^
Hibiscus	24.64 ± 2.69 ^a^	14.93 ± 1.00 ^b^	121.77 ± 9.84 ^b^
Turmeric	27.67 ± 0.42 ^a^	14.31 ± 1.53 ^b^	109.81 ± 8.10 ^b^

Differences between the values marked with the same letters in individual columns are non-significant at a 0.05 level of confidence.

**Table 4:** Water vapour barrier properties of films.

Test Conditions	Sample	WVTR (g m^−2^ h^−1^)	WVP × 10^10^ (g m^−1^ s^−1^ Pa^−1^)
RH 55%	Control	4.59 ± 0.32 ^a^	0.85 ± 0.06 ^a^
	Hibiscus	9.89 ± 0.96 ^b^	3.88 ±0.38 ^b^
	Turmeric	12.17 ± 1.12 ^c^	3.74 ± 0.34 ^b^
RH 84%	Control	30.38 ± 0.50 ^d^	3.57 ± 0.06 ^b^
	Hibiscus	30.02 ± 0.41^d^	7.43 ± 0.10 ^d^
	Turmeric	31.46 ± 1.37 ^d^	6.09 ± 0.27 c
RH 100%	Control	37.44 ± 0.54 ^e^	3.69 ± 0.05 ^b^
	Hibiscus	37.10 ± 0.91 ^e^	7.71 ± 0.19 ^d^
	Turmeric	38.06 ± 1.34 ^e^	6.19 ± 0.22 ^c^
One-way ANOVA-*p*			
		<0.001	<0.001
Two-way ANOVA-*p*			
Factor A (RH)		<0.001	<0.001
Factor B (film)		<0.001	<0.001
Factor A × Factor B		<0.001	<0.001

WVTR—water vapour transmission rate, WVP—water vapour permeance. Note: Differences between the values marked with the same letters in individual columns are non-significant at a 0.05 level of confidence. Abbreviation: ANOVA, analysis of variance.

**Table 5:** Water contact angle (°) of films.

Sample	Matte Surface		Glossy Surface	
Control	105.20 ^e^ ± 0.24	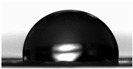	92.12 ^d^ ± 0.09	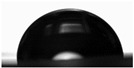
Hibiscus	87.05 ^c^ ± 1.55	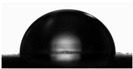	81.93 ^b^ ± 0.06	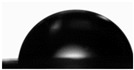
Turmeric	87.55 ^c^ ± 1.92	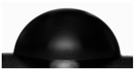	76.30 ^a^ ± 3.66	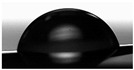

The values listed represent the mean value of three replications ± standard deviation. Mean values with the same superscripts do not differ significantly (at a level of confidence of 0.05).

**Table 6:** Melting peak parameters and exothermic transition temperature.

Sample	T_onm_ (°C)	T_pm_ (°C)	T_endm_ (°C)	−ΔH_m_ (J·g^−1^)	T_pe_ (°C)
Control	254.9 ± 13.4 ^b^	259.3 ± 13.3 ^b^	264.8 ± 13.1 ^b^	92.3 ± 15.8 ^b^	332.6 ± 4.1 ^b^
Hibiscus	244.6 ± 15.8 ^b^	248.8 ± 15.0 ^b^	254.9 ± 15.0 ^b^	86.0 ± 19.9 ^b^	321.5 ± 1.9 ^a^
Turmeric	231.8 ± 8.6 ^a^	236.5 ± 8.6 ^a^	243.0 ± 8.6 ^a^	72.1 ± 7.5 ^a^	322.4 ± 2.0 ^a^
One-way ANOVA-*p*	<0.001	<0.001	<0.001	0.007	<0.001

Mean value of at least five replications ± standard deviation. Differences between the values marked with the same letters in individual columns are non-significant at the 0.05 level of confidence. T_onm_ (onset of melting), T_pm_ (peak of melting), T_endm_ (endpoint of melting), ΔH_m_ (melting heat), T_pe_ (peak of exothermic transition).

**Table 7:** Glass transition characteristics.

Sample	T_ong_ (°C)	T_midg_ (°C)	T_infg_ (°C)	T_endg_ (°C)	ΔCp (°C)
Control	131.8 ± 10.8	143.1 ± 10.4	142.6 ± 11.3	152.3 ± 9.9	1.6 ±0.5
Hibiscus	129.4 ± 6.0	139.9 ± 6.3	138.2 ± 6.5	149.7 ± 8.1	1.6 ± 0.2
Turmeric	125.5 ± 2.8	141.6 ± 11.1	136.6 ± 6.4	149.1 ± 11.7	1.2 ± 0.1
One-way ANOVA-*p*	0.415	0.865	0.530	0.866	0.075

Mean value of at least five replications ± standard deviation. T_midg_ (midpoint of glass transition), T_infg_ (inflexion point of glass transition), T_endg_ (endpoint of glass transition), ΔCp (change in heat capacity of the transition).

**Table 8:** Results of microbiological analyses.

Sample	Cottage Cheese [cfu/g]		Salmon [cfu/g]	
	Total Bacteria Count	*E. coli*	Total Bacteria Count	*E. coli*
Fresh product	2.5 × 10^6^ ± 0.61^a^	absence	1.8 × 10^3^ ± 1.15 ^c^	3.3 × 10^2^ ± 1.53 ^b^
Without foil	2.5 × 10^6^±0.91 ^a^	absence	1.7 × 10^5^ ± 0.58 ^a^	3.5 × 10^3^ ± 0.75 ^a^
Control	2.1 × 10^6^ ± 0.55 ^a^	absence	1.3 × 10^5^ ± 1.53 ^a^	3.3 × 10^3^ ± 0.64 ^a^
Hibiscus	1.1 × 10^6^ ± 0.30 ^a^	absence	1.3 × 10^5^ ± 0.58 ^a^	2.8 × 10^3^ ± 0.55 ^a^
Turmeric	1.7 × 10^6^ ±0.53 ^a^	absence	3.0 × 10^4^ ± 2.65 ^b^	7.7 × 10^2^ ± 0.31 ^b^

The same superscript letters in each column demonstrate a lack of significant difference between values (*p* ˂ 0.05). Values are expressed as mean ± SD.

## Data Availability

The data presented in this study are available upon request from the corresponding author.
